# Identification of Alzheimer's disease using a convolutional neural network model based on T1-weighted magnetic resonance imaging

**DOI:** 10.1038/s41598-020-79243-9

**Published:** 2020-12-17

**Authors:** Jong Bin Bae, Subin Lee, Wonmo Jung, Sejin Park, Weonjin Kim, Hyunwoo Oh, Ji Won Han, Grace Eun Kim, Jun Sung Kim, Jae Hyoung Kim, Ki Woong Kim

**Affiliations:** 1 Department of Neuropsychiatry, Seoul National University Bundang Hospital, Seongnam, Korea; 2 Department of Psychiatry, Seoul National University College of Medicine, Seoul, Korea; 3 Department of Brain and Cognitive Sciences, Seoul National University College of Natural Sciences, Seoul, Korea; 4 VUNO Inc., Seoul, Korea; 5 Department of Radiology, Seoul National University Bundang Hospital, Seongnam, Korea

**Keywords:** Magnetic resonance imaging, Diagnostic markers, Alzheimer's disease

## Abstract

The classification of Alzheimer’s disease (AD) using deep learning methods has shown promising results, but successful application in clinical settings requires a combination of high accuracy, short processing time, and generalizability to various populations. In this study, we developed a convolutional neural network (CNN)-based AD classification algorithm using magnetic resonance imaging (MRI) scans from AD patients and age/gender-matched cognitively normal controls from two populations that differ in ethnicity and education level. These populations come from the Seoul National University Bundang Hospital (SNUBH) and Alzheimer’s Disease Neuroimaging Initiative (ADNI). For each population, we trained CNNs on five subsets using coronal slices of T1-weighted images that cover the medial temporal lobe. We evaluated the models on validation subsets from both the same population (within-dataset validation) and other population (between-dataset validation). Our models achieved average areas under the curves of 0.91–0.94 for within-dataset validation and 0.88–0.89 for between-dataset validation. The mean processing time per person was 23–24 s. The within-dataset and between-dataset performances were comparable between the ADNI-derived and SNUBH-derived models. These results demonstrate the generalizability of our models to different patients with different ethnicities and education levels, as well as their potential for deployment as fast and accurate diagnostic support tools for AD.

## Introduction

The rapid and accurate determination of Alzheimer’s disease (AD) based on structural magnetic resonance imaging (MRI) has garnered significant interest among researchers, owing to an incremental amount of recent studies being driven by deep learning techniques that have achieved state-of-the-art performance in various fields, including medical image analysis. In particular, convolutional neural networks (CNNs) are predominantly employed for the analysis of image data based on their ability to handle large unstructured data and to extract important features automatically^1–3^.

Structural-MRI-based CNN models for the differentiation of patients with Alzheimer’s disease (AD) and cognitively normal (CN) controls have been reported in numerous previous studies^4–9^. However, several factors in these previous studies have limited the use of their models in clinical settings. First, the populations used for development and testing were demographically biased, meaning the true generalizability of these methods to other populations is unknown. Several previous studies have used the Alzheimer’s Disease Neuroimaging Initiative (ADNI) dataset for algorithm development and validation, excluding two studies that used the Minimal Interval Resonance Imaging in Alzheimer's Disease (MIRIAD) dataset^7^ and the Australian Imaging, Biomarker, and Lifestyle Flagship Study of Ageing (AIBL) dataset^4^ for validation. However, the ADNI, MIRIAD, and AIBL datasets are all largely comprised of Caucasians with high education levels^4,7,10^. Education level^11^ and ethnicity^12,13^ are known to affect brain structures, leading to the question of how models may perform for individuals with a lower education level and/or different ethnicity. Additionally, all previous studies have used 3D images as inputs^4–9,14^, which significantly increases computational loads and limits the construction of sufficiently deep and large neural networks. The vast number of parameters introduced by using large 3D images as inputs require a huge number of layers to be able to gain the required representational power^15^; however, the construction of such a huge number of layers is typically infeasible due to limited computational resources and GPU memory. Although some studies have circumvented this problem by using 3D small patches or selected regions of interest instead of complete 3D images^5–8^, the images used were still very large. Meanwhile, several advanced networks that are commonly used as backbones in many studies take 2D images as inputs. In other words, using MRI scans as 2D images may be beneficial^16^.

In this study, we developed an AD classification algorithm based on 2D slices of T1-weighted MRI images that include AD-sensitive brain regions from two independent populations with different ethnic and demographic backgrounds. We cross-validated the results between the two populations.

## Results

Per-person preprocessing required 11.24 ± 0.59 s for the ADNI dataset and 11.88 ± 0.58 s for the SNUBH dataset. Per-person data analysis required 11.94 ± 0.59 s for the ADNI dataset and 11.97 ± 0.58 s for the SNUBH dataset. The size of input data per person was 1.58 ± 0.25 MB for the ADNI dataset and 1.70 ± 0.24 MB for the SNUBH dataset.

The models developed from the ADNI dataset exhibited a mean within-dataset area under the receiver operating characteristic (ROC) curve (AUC(i.e., area under the curve)), accuracy, sensitivity, and specificity of 0.94, 0.89, 0.88, and 0.91, respectively (Table 1), and a between-dataset AUC, accuracy, sensitivity, and specificity of 0.88, 0.83, 0.76, and 0.89, respectively (Table 2). Although the between-dataset performances of the ADNI-derived models were very good^17^, they were lower than their within-dataset performances (*t* = 5.52, *p* = 0.005 for AUC; *t* = 5.57, *p* = 0.005 for accuracy; *t* = 2.56, *p* = 0.06 for sensitivity; *t* = 0.51, *p* = 0.64 for specificity).

**Table 1:** Within-dataset testing of AD classification algorithms.

Trial	AUC	Accuracy	Sensitivity	Specificity
**ADNI**				
1st trial^a^	0.90 (0.81–0.95)	0.85 (0.77–0.93)	0.83 (0.67–0.93)	0.90 (0.75–0.97)
2nd trial^a^	0.97 (0.90–1.00)	0.91 (0.85–0.97)	0.85 (0.68–0.95)	0.96 (0.85–1.00)
3rd trial^a^	0.95 (0.88–0.99)	0.92 (0.86–0.98)	0.94 (0.81–0.99)	0.91 (0.77–0.97)
4th trial^a^	0.95 (0.87–0.99)	0.89 (0.81–0.96)	0.97 (0.85–1.00)	0.82 (0.67–0.92)
5th trial^a^	0.95 (0.87–0.99)	0.89 (0.81–0.96)	0.81 (0.65–0.92)	0.95 (0.84–0.99)
Mean (SD)	0.94 (0.03)	0.89 (0.03)	0.88 (0.07)	0.91 (0.06)
**SNUBH**				
1st trial^a^	0.94 (0.87–0.98)	0.92 (0.86–0.98)	0.92 (0.79–0.98)	0.93 (0.80–0.98)
2nd trial^a^	0.88 (0.79–0.94)	0.82 (0.74–0.91)	0.79 (0.64–0.89)	0.87 (0.70–0.96)
3rd trial^a^	0.87 (0.77–0.94)	0.85 (0.77–0.93)	0.83 (0.66–0.93)	0.86 (0.72–0.95)
4th trial^a^	0.90 (0.81–0.96)	0.87 (0.80–0.95)	0.84 (0.69–0.93)	0.91 (0.77–0.98)
5th trial^a^	0.94 (0.86–0.98)	0.94 (0.88–0.99)	0.88 (0.73–0.97)	0.98 (0.77–0.98)
Mean (SD)	0.91 (0.03)	0.88 (0.05)	0.85 (0.05)	0.91 (0.05)
**Statistics**^**b**^				
*T*	1.93	0.40	0.72	− 0.14
*P* value	0.09	0.70	0.49	0.89

AD classification algorithms were developed by randomly selecting 80% of the participants (156 AD patients and 156 CN controls) in each dataset (ADNI and SNUBH) and tested within each dataset on the remaining 20% of the participants (39 AD patients and 39 CN controls).

*AUC* area under the receiver operating characteristic curve, *ADNI* dataset from the Alzheimer’s Disease Neuroimaging Initiative, *SNUBH* dataset from the Seoul National University Bundang Hospital, *SD* standard deviation.

^a^95% confidence intervals in parentheses.

^b^Comparison of performances on the ADNI and SNUBH datasets using Student’s t-test.

**Table 2:** Between-dataset testing of AD classification algorithms.

Trial	AUC	Accuracy	Sensitivity	Specificity
**ADNI**				
1st trial^a^	0.87 (0.84–0.90)	0.82 (0.78–0.86)	0.80 (0.74–0.85)	0.84 (0.78–0.89)
2nd trial^a^	0.88 (0.85–0.91)	0.83 (0.79–0.87)	0.77 (0.70–0.83)	0.89 (0.83–0.93)
3rd trial^a^	0.88 (0.84–0.91)	0.83 (0.79–0.87)	0.74 (0.67–0.80)	0.92 (0.88–0.96)
4th trial^a^	0.89 (0.86–0.92)	0.84 (0.80–0.87)	0.74 (0.68–0.80)	0.93 (0.88–0.96)
5th trial^a^	0.86 (0.82–0.89)	0.81 (0.77–0.85)	0.76 (0.69–0.82)	0.86 (0.80–0.90)
Mean (SD)	0.88 (0.01)	0.83 (0.01)	0.76 (0.03)	0.89 (0.04)
**SNUBH**				
1st trial^a^	0.90 (0.87–0.93)	0.83 (0.79–0.86)	0.85 (0.79–0.90)	0.80 (0.74–0.85)
2nd trial^a^	0.88 (0.84–0.91)	0.81 (0.77–0.85)	0.72 (0.65–0.78)	0.90 (0.85–0.94)
3rd trial^a^	0.90 (0.86–0.92)	0.82 (0.79–0.86)	0.79 (0.73–0.85)	0.86 (0.80–0.90)
4th trial^a^	0.89 (0.85–0.92)	0.83 (0.79–0.87)	0.82 (0.75–0.87)	0.84 (0.78–0.89)
5th trial^a^	0.88 (0.84–0.91)	0.80 (0.76–0.84)	0.77 (0.71–0.83)	0.83 (0.77–0.88)
Mean (SD)	0.89 (0.01)	0.82 (0.01)	0.79 (0.05)	0.85 (0.04)
**Statistics**				
ADNI—SNUBH^b^				
*t*	− 1.53	1.01	− 0.79	1.64
*P* value	0.17	0.34	0.45	0.14
Within—between^c^				
ADNI				
*t*	5.52	5.57	2.56	0.51
*P* value	0.005	0.005	0.06	0.64
SNUBH				
*t*	1.26	2.64	4.17	1.86
*P* Value	0.28	0.06	0.01	0.14

AD classification algorithms were developed by randomly selecting 80% of the participants (156 AD patients and 156 CN controls) in each dataset (ADNI and SNUBH) and tested within each dataset on the remaining 20% of the participants (39 AD patients and 39 CN controls).

*AUC* area under the receiver operating characteristic curve, *ADNI* dataset from the Alzheimer’s Disease Neuroimaging Initiative, *SNUBH* dataset from the Seoul National University Bundang Hospital, *SD* standard deviation.

^a^95% confidence intervals in parentheses.

^b^Comparison of performances on the ADNI and SNUBH datasets using Student’s t-test.

^c^Comparison of within-dataset and between-dataset performances using a paired t-test.

The models developed from the SNUBH dataset exhibited a mean within-dataset AUC, accuracy, sensitivity, and specificity of 0.91, 0.88, 0.85, and 0.91, respectively (Table 1), and a between-dataset AUC, accuracy, sensitivity, and specificity of 0.89, 0.82, 0.79, and 0.85, respectively (Table 2). The between-dataset performances of the SNUBH-derived models were also very good^17^. The between-dataset and within-dataset AUC, accuracy, and specificity were comparable, but the between-dataset sensitivity was slightly lower than the within-dataset sensitivity (*t* = 1.26, *p* = 0.28 for AUC; *t* = 2.64, *p* = 0.06 for accuracy; *t* = 4.17, *p* = 0.01 for sensitivity; *t* = 1.86, *p* = 0.14 for specificity).

The within-dataset performances (*t* = 1.93, *p* = 0.09 for AUC; *t* = 0.40, *p* = 0.70 for accuracy; *t* = 0.72, *p* = 0.49 for sensitivity; *t* = − 0.14, *p* = 0.89 for specificity) and between-dataset performances (*t* = − 1.53, *p* = 0.17 for AUC; *t* = 1.01, *p* = 0.34 for accuracy; *t* = − 0.79, *p* = 0.45 for sensitivity; *t* = 1.64, *p* = 0.14 for specificity) were comparable between the ADNI-derived and SNUBH-derived models.

## Discussion

In this study, we propose a CNN-based algorithm that uses MRI coronal slices covering the medial temporal lobe to classify AD patients and CN controls. We trained and validated our algorithm on two independent populations with different ethnicities and education levels. Experimental results demonstrate that our algorithm is fast and can provide high accuracy, regardless of the ethnic and/or demographic characteristics of subjects.

Our algorithm considers the medial temporal lobe atrophy (MTA) scale, which is widely used in clinical practice to determine the presence of AD-related neurodegeneration. This scale is also used as neurodegenerative evidence for AD according to the National Institute on Aging and Alzheimer’s Association research guidelines/framework^18^. Although other regions may also provide useful information for AD classification, there is known to be slight inter-subject variability in exact atrophy patterns^19^ and medial temporal lobe (MTL)-focused atrophy is the most common type. Therefore, attempting to add other regions may confuse the algorithm and result in potential misclassification with other diseases that share atrophy patterns in similar areas. Another reason we selected the 30 coronal slices is to cover the entire length of the hippocampus and to give additional weight/attention to those slices because that area contains the most essential information. The robust performance of the proposed algorithm in our experimental results suggests that the assigned weights were appropriate.

To the best of our knowledge, ours is the first CNN-based AD classification algorithm that uses 2D images as input data. The accuracy of our ADNI-based models (AUC = 0.890) is greater than those of previous deep learning methods that also used ADNI data^20–23^. Although using 2D images as inputs for a neural network may provide less information compared to 3D images or 3D patches, we were able to construct a network containing 487 layers, which is much deeper than previous 3D-image-based CNNs (≤ 39 layers), allowing it to learn more complicated representations. Using 2D images as input data has several practical advantages. First, such images are more widely applicable in clinical settings, where 3D MRI scans may not always be available. Second, it can reduce processing time and computational resources significantly for implementation in clinical settings, where clinicians are typically pressed for time and computational resources are limited. CNN models that take 2D data as inputs have lower computational complexity and lower memory bandwidth demands than 3D CNN models^24^. Finally, there are numerous public datasets of 2D images, such as ImageNet, CIFAR, Birdsnap, Stanford Cars, and Oxford-IIIT Pets, which can contribute to the rapid advancement and development of novel 2D CNN architectures^16^. Therefore, using 2D MRI slices allows us to apply the latest CNN architectures.

To the best of our knowledge, all previous studies that have validated AD classification models have considered nearly homogenous ethnic populations, typically consisting of Caucasians, and no cross-ethnicity investigations have been performed. This introduces several potential biases into the evaluation of a model’s true accuracy and robustness—brain shape is known to be variant across ethnicities, with the brains of Asians being wider and shorter than those of Caucasians^12^. Agreement between clinical and pathological diagnoses of AD also differs between ethnicities, with agreement being 90% for Caucasians^25^ but only 34% for Japanese Americans^26^. Additionally, demographic characteristics such as education level are known to alter AD-associated structural brain changes^11^. Therefore, to assess the true generalizability and practical utility of MRI-based AD classification algorithms, it is important to cross-validate an algorithm that is trained on one population on other populations with different ethnic and demographic characteristics. In this study, for the first time, we directly cross-validated models trained on a population that consists mainly of highly educated Caucasians and a population that consists mainly of moderately educated Asians. We found that the between-dataset performances of both the ADNI-derived and SNUBH-derived models exhibited good accuracy (AUC = 0.88 and 0.89, respectively) and were not affected by the population used for training (*p* = 0.17). In other words, when our model is trained on population A, it is able to perform well on population B, and vice versa (at least for Caucasians and Asians). The consistent performances of our algorithm on both populations suggest that deep learning models using MRI images can be transferrable across populations of different ethnicities. This can be attributed to the fact that the signature atrophy patterns of AD (mainly in the hippocampus and MTL structures) are consistent across different ethnic populations, including Caucasians^19^, Asians^27^, and Africans^28^. This consistency may give MRI-based algorithms an advantage in being able to generalize from one ethnic population to another, whereas AD classification methods based on neuropsychiatric tests may have limitations in generalizability based on underlying ethnic differences in terms of language, educational level, and culture.

There are several limitations in this study that must be addressed. One limitation is that the MRI images from SNUBH were acquired using scanners from a single manufacturer (Philips), whereas the MRI images from ADNI were obtained using various scanners (Siemens, GE, and Philips) with different MRI protocols. This may have contributed to the result that the between-dataset performances of the models tended to be lower than the within-dataset performances. Future studies should investigate the effects of scanners and/or scanning protocols on the diagnostic accuracy of deep-learning-based models. Additionally, our dataset did not include individuals with mild cognitive impairment (MCI), which are considered to be high-risk individuals for dementia. However, MCI is a pathologically heterogeneous group with multiple etiologies and causes^29^, with approximately 50% having AD pathology^30^. Because we were not able to check for the presence of AD pathology in this study (which would require amyloid positron emission tomography (PET) scans), we did not include MCI in our model because this could potentially offset the accuracy of the model. Although we were unable to utilize MCI due to AD in our model construction or evaluate our model on MCI due to AD, we believe that when used in a well-designed experiment on PET-confirmed MCI patients due to AD, our model will still show a satisfactory performance. This is because we only considered mild AD patients with a clinical dementia rating (CDR) score of 0.5 to 1.

In this study, we developed and extensively validated an AD classification CNN-based algorithm using two independent populations. Our approach using 2D slices corresponding to the early neurodegenerative sites of AD has practical advantages in terms of both processing speed and accuracy, regardless of a subject’s demographic characteristics.

## Methods

### Datasets

We used two datasets in this study: one from the ADNI and the other from the SNUBH. From ADNI, we included participants in both ADNI1 and ADNI2 who had 3.0 T T1-weighted images and were diagnosed as CN or mild AD (CDR of 0.5 or 1). For up-to-date information about the ADNI, see https://www.adni-info.org. From the SNUBH, we included AD patients and CN controls with T1-weighted images whose age, sex, and CDR were matched to the patients from the ADNI. However, we were unable to further match for education and cognitive level because participants from the ADNI were more educated and performed better on the Mini Mental State Examination (MMSE) than those from the SNUBH. In the case where a participant has multiple MRI scans from different timepoints, we selected only one MRI scan based on the participant’s age and diagnosis at the time of assessment. We selected the scan whose demographic factors would contribute to a more demographically balanced dataset. In terms of ethnicity, the ADNI dataset contained Caucasians (83.59%), African-Americans (4.87%), Hispanics (5.64%), Asians (2.05%), and others (3.85%), while the SNUBH dataset contained only Koreans (Table 3).

**Table 3:** Characteristics of participants.

	AD				CN			
	ADNI	SNUBH	t or χ^2^	*P* Value	ADNI	SNUBH	t or χ^2^	*P* value
N	195	195	-	-	195	195	–	–
Age (years, mean ± SD)	74.7 ± 8.2	74.5 ± 8.7	0.2	0.84	74.8 ± 6.6	73.9 ± 6.3	1.4	0.17
Sex (women, %)	91 (46.7%)	91 (46.7%)	0.0	> 0.99	91 (46.7%)	91 (46.7%)	0.0	> 0.99
Education (years, mean ± SD)	15.5 ± 2.9	10.2 ± 5.4	12.2	< 0.001	16.0 ± 2.7	11.4 ± 4.8	11.6	< 0.001
CDR (score, mean ± SD)	0.8 ± 0.3	0.8 ± 0.3	0.0	> 0.99	0.0 ± 0.0	0.0 ± 0.0	0.0	> 0.99
SOB (score, mean ± SD)	4.5 ± 1.6	4.3 ± 1.9	1.0	0.30	0.0 ± 0.0	0.0 ± 0.0	0.0	> 0.99
MMSE (score, mean ± SD)	23.0 ± 2.3	18.4 ± 5.0	11.7	< 0.001	29.1 ± 1.3	27.2 ± 2.4	9.5	< 0.001

*AD* Alzheimer’s disease, *CN* cognitively normal, *ADNI* Alzheimer’s Disease Neuroimaging Initiative, *SNUBH* Seoul National University Bundang Hospital, *SD* standard deviation, *CDR* clinical dementia rating scale, *SOB* sum of box scores of CDR, *MMSE* Mini Mental State Examination.

The protocol for this study was approved by the Institutional Review Board of the SNUBH. We acquired written informed consent from the subjects or their legal guardians. The ADNI was approved by the institutional review board at each site and all participants gave their written consent. All procedures were performed in accordance with the relevant guidelines and regulations.

### Diagnostic criteria

In both the ADNI and SNUBH, AD was diagnosed according to the National Institute of Neurological and Communicative Disorders and Stroke, and the Alzheimer’s Disease and Related Disorders Association criteria for probable AD^31^. CN was defined by the absence of subjective cognitive complaints and a normal score on cognitive tests. Normal scores on cognitive tests were defined differently in each population, owing to the fact that the populations used a different set of tools for objective cognitive evaluation. In the ADNI, the Logical Memory II subscale of the Wechsler Memory Scale-Revised score was used, with scores of > 8, > 4, and > 2 for > 16, 8–15, and 0–7 years of education, respectively, indicating normal cognition. In SNUBH, the Consortium to Establish a Registry for Alzheimer’s Disease Korean version was used, with standard deviations (SDs) greater than − 1.5 for the age-, sex-, and education-adjusted norms on ten neuropsychological tests (i.e., Categorical Fluency Test, modified Boston Naming Test, Word List Memory Test, Constructional Praxis Test, Word List Recall Test, Word List Recognition Test, Constructional Recall Test, Trail Making Test A, Digit Span Test, and Frontal Assessment Battery) indicating normal cognition^32^.

### Image acquisition and preprocessing

For the ADNI dataset, 3D T1-weighted MRI scans were acquired in digital imaging and communications in medicine (DICOM) format using Siemens (49.23%), GE (29.74%), and Philips (21.03%) scanners (details regarding the ADNI MRI data acquisition protocol can be found on ADNI's official webpage: adni.loni.usc.edu). For the SNUBH dataset, we acquired 3D structural T1-weighted MRI images in DICOM format using Philips scanners only (voxel dimensions = 1.0 × 0.5 × 0.5 mm, slice thickness = 1.0 mm, echo time = 8.15 or 8.20 ms, repetition time = 4.61 ms, flip angle = 8°, field of view = 240 × 240 mm).

**Figure 1:**
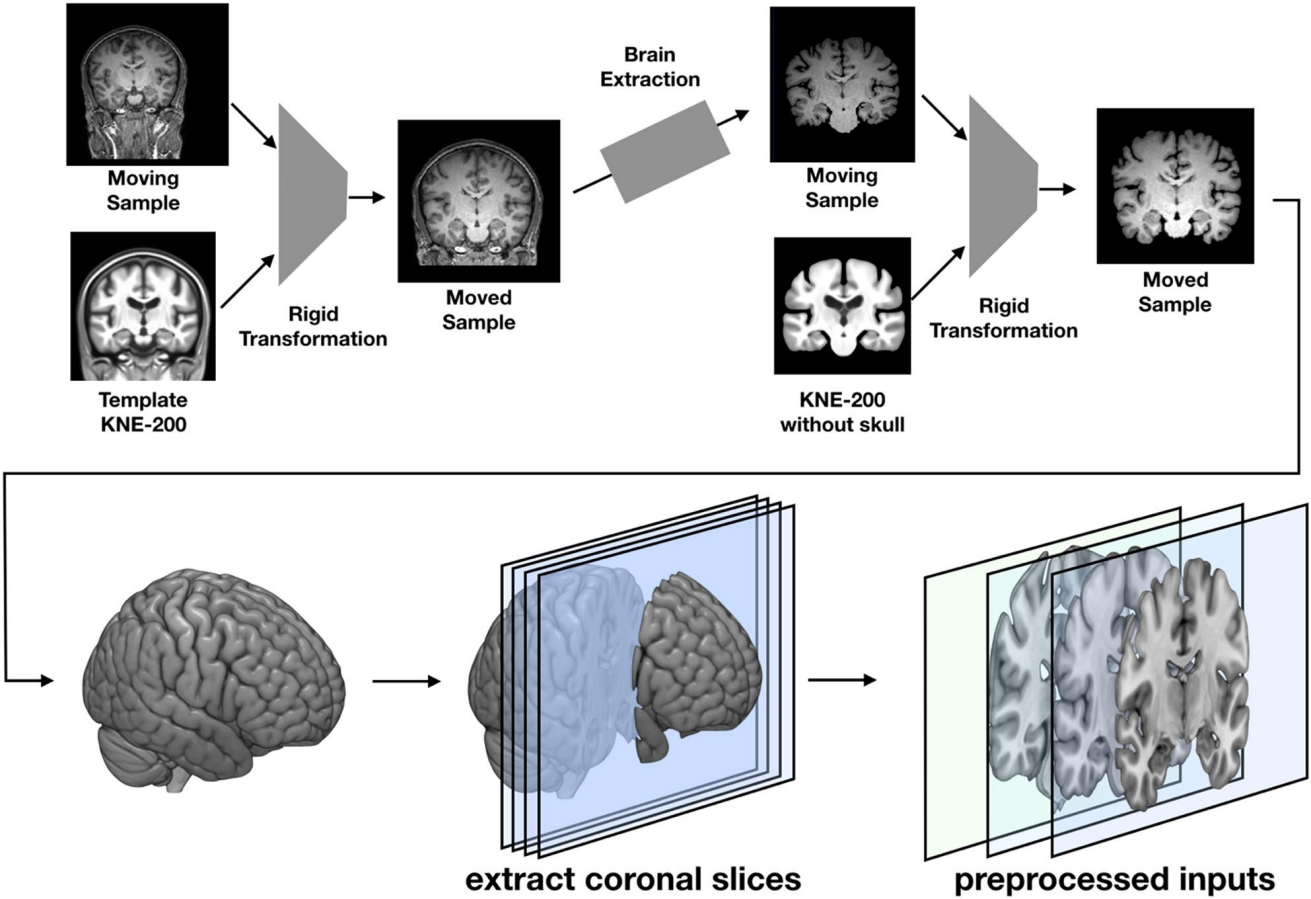
Preprocessing for extracting 2D coronal slices of the medial temporal lobe from complete 3D brain scans. The input whole-brain 3D T1-weighted MRI images are subjected to an initial rigid transformation to fit a template, followed by brain extraction (skull stripping). Next, a second rigid transformation is applied to the skull-stripped version of the template. Once the subject image is in the same space as the template, the range of slices that correspond to the MTL in the template are used to extract coronal slices from the template-registered output subject image.

The 3D T1-weighted brain image inputs were first resampled into a grid of 256 × 256 × 256 voxels with an isotropic spatial resolution of 1 × 1 × 1 mm using the mri_convert routine in FreeSurfer^33^. From the resampled complete images, coronal slices around the MTL were extracted using two rounds of rigid transformation (Fig. 1). In the first rigid transformation, the position of the input image was matched to a template constructed from a CN elderly population^12^. The template-registered input image was then processed by a custom brain extraction algorithm to extract only the brain parenchyma. The custom brain extraction algorithm is based on a 3D UNet trained to extract brain parenchyma using labels generated by the Brain Extraction Tool in FMRIB Software Library^34^. In the second rigid transformation, the skull-stripped and template-registered input images were registered to a skull-stripped version of the template from the first step (skull-stripped using the same custom algorithm). In this manner, the two-step rigid transformation process was used to increase the accuracy of the registration of each subject’s brain parenchyma to the template. Rigid transformation, which was performed using the Advanced Normalization Tools library, was used to avoid changing the morphological structure of the brain parenchyma^35^.

Next, 2D coronal slices were extracted from the output images from the second rigid transformation. Among the 256 coronal slices, 30 coronal slices starting from the corpus of the hippocampus (at the level of the anterior pons) were extracted. These slices were selected based on the criteria used for conventional coronal slice selection in the MTA visual rating scale^36^. In each slice, min–max normalization was applied to bound the values of the images between zero and one.

### Deep learning model

**Figure 2:**
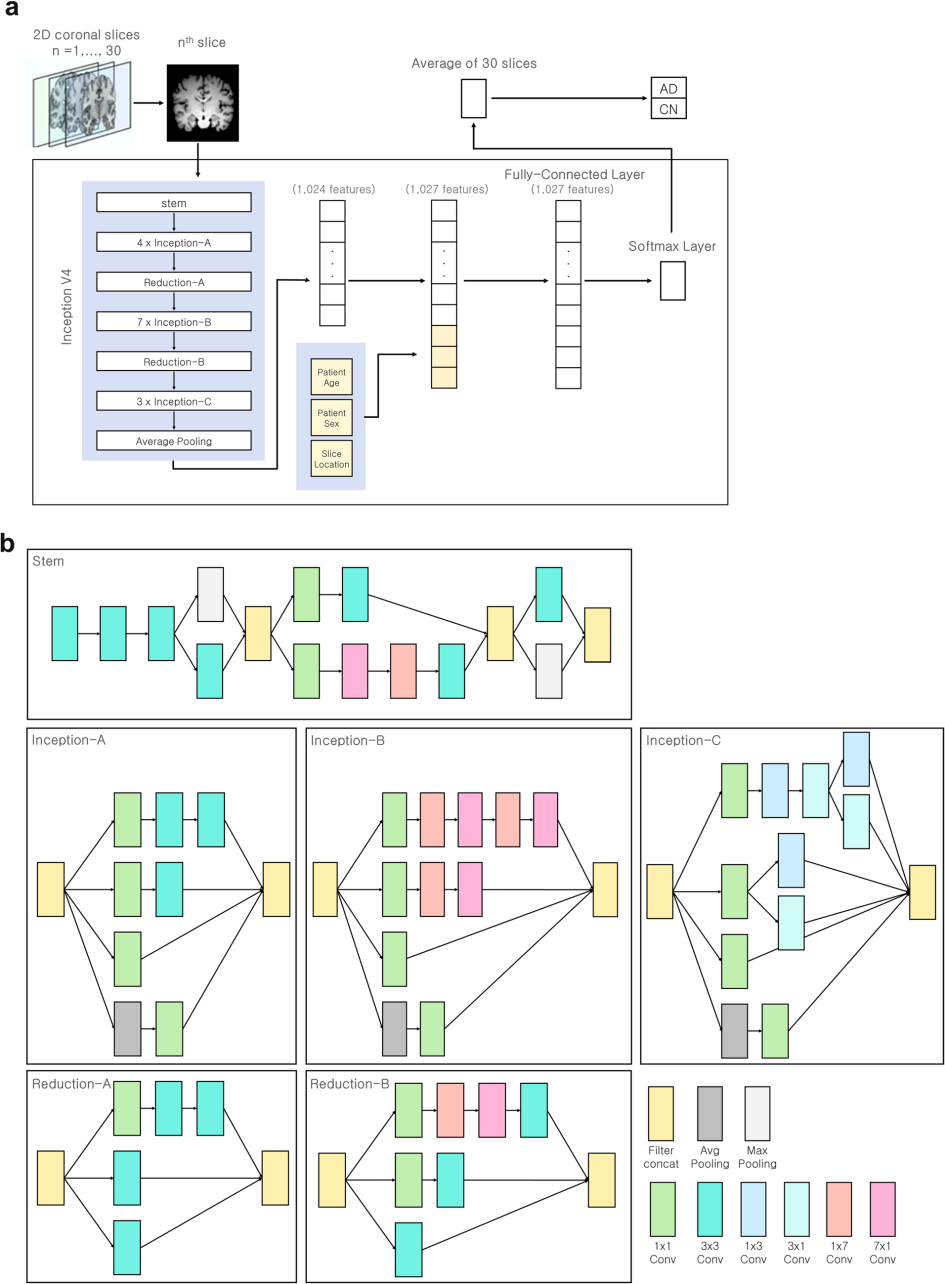
Diagram of the network architecture. For each subject, 1 out of 30 coronal slices are fed into the model independently, and the results of the 30 slices are averaged to produce a AD probability for that subject. The first part of the model consists of the architecture of a pretrained network (Inception V4), and the last part of the model involves the addition of the subject’s age, sex, and slice location **(a)**. The specific constituents of Inception v4 are shown (stem, Inception-A, Inception-B, Inception-C, Reduction-A, Reduction-B) **(b).**

Each of the preprocessed coronal slices were then fed individually into our neural network and the outputs for each slice were averaged to perform classification for the corresponding subject. For the neural network, we used the Inception-v4 architecture as a backbone with a few modifications^37^. Inception-v4 is a 2D image classification neural network that has been shown to achieve very good performance with low computational cost. We also adopted its pre-trained weights (https://github.com/Cadene/pretrained-models.pytorch), which were obtained from a subset of ImageNet used for a classification task in the ImageNet Large-Scale Visual Recognition Competition in 2012, which is a training dataset containing 1.28 million natural images from 1000 categories^37,38^. The Inception-v4 architecture was designed to take 2D images with three RGB channels as inputs. Therefore, we triplicated our greyscale coronal slices into three channels for consistency. After a single coronal slice was entered into the Inception backbone architecture, a feature vector containing 1024 values representing the results of convolution was produced. We then added three additional values to the end of the vector (subject age, sex, and the number of coronal slice being evaluated). We added the subject age because mild MTA is observed in CN elderly individuals, that is the magnitude of atrophy should be evaluated with reference to the subject’s age. The final concatenated feature vector containing 1027 values was then fed into the final classifier module. The classifier module of Inception-v4 was replaced with a fully connected layer with 1027 input nodes and two output nodes. Finally, the output of the fully connected layer was fed into a softmax output layer to predict the probability that an input brain MR image indicates the presence of AD. Our CNN model contains a total of 497 layers. The architecture of our model is illustrated in Fig. 2.

AD classification is a binary classification problem for predicting the presence of AD. Each slice image is labeled as AD or CN and the results for all slices are averaged. The inputs are 2D coronal slices x_i_ from a patient’s 3D MRI brain scan x, and the output is y, which is a value indicating the probability of the presence of AD. During training, the binary cross-entropy loss of the predicted outputs for a single batch is calculated as follows:

$$J\left(w\right)=-\frac{1}{N}\sum_{n=1}^{N}[{y}^{n}log\left(f\left({x}_{i}^{n};w\right)\right){\widehat{y}}^{n}+(1-{y}^{n})\mathrm{log}(1-f({x}_{i}^{n};w))],$$

where x_i_ is a slice from patient x, y_i_ is a true class of x_i_, and N is the number of samples in a single batch. During validation and testing, the averaged probability of all input slices (x_1_, x_2_, …, x_n_) for patient x was used as the final predicted probability of the presence of AD. All models were optimized using mini-batch stochastic gradient descent with Nesterov momentum^39^ and a batch size of 64 to maximize GPU utilization. We used a weight decay of 5 × 10^−5^ and base learning rate of 0.001, which decayed by 0.1 three times until the validation loss plateaus.

Real-time data augmentation was performed to make our models learn features that remained invariant under geometric and intensity perturbations. Rotation, scaling, translation, contrast changes, and gamma adjustment were applied for data augmentation. All parameters for the data augmentation operations were randomly selected from predefined ranges.

All experiments were conducted using NVIDIA 1080ti GPUs with 11 GB of memory per GPU and all deep learning models were implemented using Pytorch (v.0.4.1). We performed stratified fivefold cross-validations to distribute samples equally by considering class balance between the training set and validation set. In each fold, we terminated training if the moving average of validation accuracy did not improve by more than 5 × 10^4^ within the last 5 epochs.

**Figure 3:**
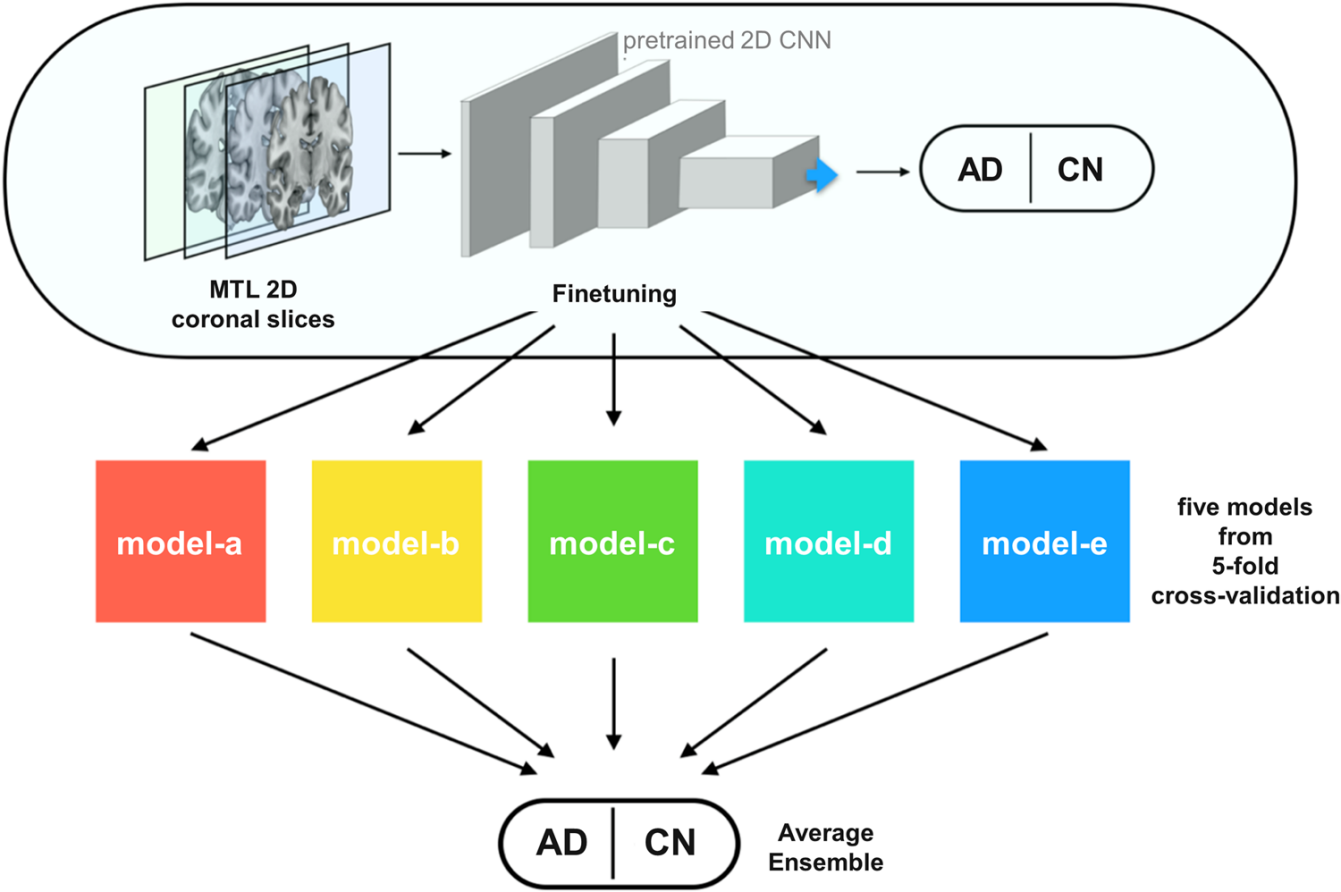
Training of the coronal slice-based AD classification model. We performed stratified fivefold cross-validation to distribute the samples equally by considering class balance between the training set and validation set. The average ensemble values of the average probabilities of the models (models a to e) generated from cross-validation are then used as the final results in the testing phase.

To predict the class of a subject during validation and testing, we used the average of the predicted probabilities for each of the 30 slices extracted from that subject. The final prediction values for the test sets were estimated from the average ensemble values of the five runs of fivefold cross-validation for the development set (Fig. 3).

### Statistical analysis

For each dataset (ADNI and SNUBH), we randomly divided the MRI scans from 390 participants and assigned 80% for development and 20% for testing (randomization). This resulted in a development set containing 156 AD and 156 CN patients, and a test set containing 39 AD and 39 CN patients for each dataset. We trained an AD classification model using the 80% development set and tested it using the 20% testing set for the ADNI and SNUBH datasets separately (within-dataset testing). We then retested the AD classification model trained on the ADNI development set on the entire SNUBH dataset and that trained on the SNUBH development set on the entire ADNI dataset (between-dataset testing). We repeated this process five times for each of the ADNI and SNUBH datasets (five trials).

We tested the performance of the algorithms based on ROC curve analyses. For model evaluation, we measured the AUC, accuracy, sensitivity, and specificity of each model for each test dataset. We derived sensitivity and specificity values according to Youden’s index^40^ and calculated accuracy by counting the number of true positive and true negative cases at the optimal associated criterion according to the Youden’s index and dividing the result by the total number of cases. We compared the AUCs of the algorithms developed from the two datasets using the DeLong test^41^ and compared accuracy, sensitivity, and specificity using Student’s t-test. We compared continuous variables based on independent samples using Student’s t-test or a paired t-test as appropriate and compared categorical variables using the chi-squared test. We considered two-sided *p-*values less than 0.05 to be statistically significant.

We performed statistical analyses using SPSS version 20 (SPSS, Inc., Chicago, IL, USA) and MedCalc version 16.4.3 (MedCalc Software, Mariakerke, Belgium).

## Data Availability

The data that support the findings of this study are available from the authors upon reasonable request.
